# Betulinic acid inhibits pyroptosis in spinal cord injury by augmenting autophagy via the AMPK-mTOR-TFEB signaling pathway

**DOI:** 10.7150/ijbs.57825

**Published:** 2021-03-11

**Authors:** Chenyu Wu, Huanwen Chen, Rong Zhuang, Haojie Zhang, Yongli Wang, Xinli Hu, Yu Xu, Jiafeng Li, Yao Li, Xiangyang Wang, Hui Xu, Wenfei Ni, Kailiang Zhou

**Affiliations:** 1 Department of Orthopaedics, The Second Affiliated Hospital and Yuying Children's Hospital of Wenzhou Medical University, Wenzhou 325027, China.; 2 Zhejiang Provincial Key Laboratory of Orthopaedics, Wenzhou 325027, China.; 3 University of Maryland School of Medicine, Baltimore, MD 21201, USA.; 4 Department of Anesthesiology, Critical Care and Pain Medicine, The Second Affiliated Hospital and Yuying Children's Hospital of Wenzhou Medical University, Wenzhou 325027, China.; 5 Department of Orthopaedics, Huzhou Central Hospital, Huzhou 313000, China.

**Keywords:** Betulinic acid, autophagy, mitophagy, pyroptosis, spinal cord injury

## Abstract

Spinal cord injury (SCI) results in a wide range of disabilities. Its complex pathophysiological process limits the effectiveness of many clinical treatments. Betulinic acid (BA) has been shown to be an effective treatment for some neurological diseases, but it has not been studied in SCI. In this study, we assessed the role of BA in SCI and investigated its underlying mechanism. We used a mouse model of SCI, and functional outcomes following injury were assessed. Western blotting, ELISA, and immunofluorescence techniques were employed to analyze levels of autophagy, mitophagy, pyroptosis, and AMPK-related signaling pathways were also examined. Our results showed that BA significantly improved functional recovery following SCI. Furthermore, autophagy, mitophagy, ROS level and pyroptosis were implicated in the mechanism of BA in the treatment of SCI. Specifically, our results suggest that BA restored autophagy flux following injury, which induced mitophagy to eliminate the accumulation of ROS and inhibits pyroptosis. Further mechanistic studies revealed that BA likely regulates autophagy and mitophagy via the AMPK-mTOR-TFEB signaling pathway. Those results showed that BA can significantly promote the recovery following SCI and that it may be a promising therapy for SCI.

## Introduction

Spinal cord injury (SCI) is a disabling disease that often results in significant motor and sensory dysfunctions 1, and it affects over 500,000 patients in the United States alone 2. Currently, there are only a few therapeutic interventions for SCI, with high-dose methylprednisolone 3, ganglioside 4, immunoglobulin G 5, and exogenous basic fibroblast growth factor 6 showing some clinical benefit in select patients; however, these agents have largely been unable to improve recovery following SCI. The dearth of therapeutic options for SCI is likely due to its complex pathophysiology. In brief, SCI is thought to involve two distinct injury mechanisms: primary and secondary. Primary injury occurs as a direct consequence of mechanical damage of the spinal cord, whereas secondary injury is triggered by a delayed sequence of complex biochemical and cellular processes such as oxidative stress accumulation, inflammation 7, apoptosis, programmed cell death, and various other processes 8. Together, primary and secondary injuries lead to irreversible neuronal damage, which ultimately culminates in poor functional recovery of patients following SCI 9. Thus, preventing irreversible damage by targeting pathways of programmed cell death such as apoptosis, pyroptosis, and autophagy may be a promising strategy for treating SCI 10-12.

Pyroptosis, an inflammatory form of programmed cell death, is known to play a detrimental role in acute central nervous system (CNS) injuries such as traumatic brain injury, cerebral ischemia, and SCI 13-15. Morphologically, pyroptosis is characterized by cell swelling, rupture, and release of cytoplasmic contents 16. Mechanistically, following CNS injury, inflammasomes (such as NLRP3, ASC, Caspase-1) are activated to cleave GSDMD and release proinflammatory cytokines (interleukin-1β and 18), ultimately leading to pyroptosis 16-18. In addition, extensive studies have demonstrated that a series of pathological changes such as hemorrhage, hypoxia, and edema after SCI can cause accumulations of reactive oxygen species (ROS), which can act as secondary messengers to induce NLRP3 inflammasome-mediated pyroptosis 19, 20.

Autophagy, key cellular process for removing cytosolic waste and damaged organelles 21, 22, plays a key role in the pathophysiology of neurological diseases 23. Overall, activation of autophagy is considered protective in SCI, which can attenuate pyroptosis 24 and improve functional recovery 25. More specifically, increased autophagy stimulates mitophagy (autophagic degradation of damaged mitochondria), restricts the production of ROS, dampen the activation of NLRP3 inflammasomes, and suppress the secretion of IL-1β and IL-18, ultimately inhibiting pyroptosis 22, 26, 27. Therefore, autophagy augmentation may be a viable strategy to suppress pyroptosis and improve functional recovery following SCI.

Betulinic acid (BA), a natural pentacyclic triterpenoid, is known to be found in Chinese herbal medicine extensively 28. More importantly, BA has the ability to cross the blood-brain barrier, which is of great significance in the treatment of CNS pathologies 29, 30. Past studies have shown that BA exerts neuroprotective effects via promoting autophagy 31, reducing ROS 32 and inhibiting inflammation 33. However, whether BA is an effective treatment for SCI remains unclear. Furthermore, little is known regarding BA's impact on pyroptosis. Thus, in this study, we assessed whether BA can enhance autophagy, inhibit pyroptosis, and promote functional recovery following SCI.

## Materials and methods

### Animals

Wenzhou Medical University's Experimental Animal Center (Zhejiang Province, China) supplied female C57BL/6 mice (20-30 g, license no. SCXK [ZJ] 2005-0019). All laboratory procedures involving animals followed the China National Institutes of Health's Guide for the Care and Use of Laboratory Animals, and were approved by Wenzhou Medical University's Animal Research Committee (wydw2017-0022). Best efforts were undertaken to minimize the number of animals used and pain inflicted. Standard experimental cages with a 12-h light/dark cycle were applied to house all mice individually, and unrestricted access to food and water was provided. 105 mice were divided randomly into five groups: Sham (n = 25), SCI (n = 25), Betulinic Acid (BA, n = 25), BA +3-methyladenine (BA+3MA, n = 25) and BA+ Compound C (BA+CC, n = 5).

### Reagents and antibodies

All reagents, antibodies and their suppliers are listed as follows: Betulinic Acid (C30H48O3, purity ≥98%) was obtained from MedChemExpress (Shanghai, China). HE staining kit, Masson staining kit, Diaminobenzidine (DAB) developer, and pentobarbital sodium, were purchased from Solarbio Science & Technology (Beijing, China). Dorsomorphin (Compound C, C24H25N5O; purity ≥ 98.14%) was purchased from Med Chem Express (Monmouth Junction, NJ, USA). Primary antibody against TFEB was obtained from Bethyl Laboratories (Montgomery, TX, USA). Rabbit monoclonal anti-GAPDH was purchased from Biogot Technology (Shanghai, China). The rabbit monoclonal anti-Caspase-1, anti-phosphoinositide- 3-kinase (Vps34), and anti-cathepsin D (CTSD) antibodies were purchased from Proteintech Group (Chicago, IL, USA). Rabbit monoclonal 3-methyladenine (3MA) and anti-microtubule-associated 1 protein light chain 3 (LC3) antibodies were obtained from Sigma-Aldrich Chemical Co (Milwaukee, WI, USA). NLRP3, ASC, AMPK, Beclin1, mTOR, p-AMPK and p-mTOR were obtained from Cell Signaling Technology (Beverly, MA, USA). Mouse monoclonal Synaptophysin, anti-SQSTM1/ p62, NeuN, goat anti-rabbit IgG H&L (Alexa Fluor® 488), MAP2 and goat anti-mouse IgG H&L (Alexa Fluor® 647) antibodies were acquired from Abcam (Cambridge, UK). IL-1β. Antibodies against IL-18, Bnip3, GSDMD, Nix and Parkin were obtained from Affinity Biosciences (OH.USA). Santa Cruz Biotechnology (Dallas, TX, USA) supplied the secondary antibody Horseradish peroxidase (HRP)-conjugated immunoglobulin G (IgG). Beyotime Biotechnology (Jiangsu, China) provided the 4′,6-diamidino- 2-phenylindole (DAPI), and Boyun Biotechnology (Nanjing, China) supplied the Fluorescein isothiocyanate (FITC)-conjugated IgG secondary. ThermoFisher Scientific (Rockford, IL, USA) provided the BCA kit. PerkinElmer Life Sciences (Waltham, MA, USA) supplied the Electrochemiluminescence (ECL) Plus Reagent Kit.

### Animal model of SCI

Prior to procedure, anesthesia was administered to all animals with intraperitoneal injections of one percent (w/v) pentobarbital sodium (50 mg/kg). Then, standard laminectomy was performed at the T11-T12 vertebra to expose a circle of the dura. Then, a weight-drop injury model was employed to cause a spinal contusion injury as previously described 34. In brief, a bar with a diameter of 3.0mm and a weight of 15g was dropped onto the exposed spinal cord from a 15 mm height. After injury, muscle, fascia, and skin was closed in layers using 4-0 nonabsorbable silk sutures. Mice in the sham group received the same operation as above without weight-drop injury. Following the procedure, all mice had their bladders artificially emptied three times a day.

### Drug administration

All drugs were dissolved in 2% DMSO in normal saline and administered intraperitoneally. Daily injections of 20 mg/kg BA after SCI were administered to the BA group. 3MA (15 mg/kg) and Dorsomorphin (Compound C, 1.5 mg/kg) were injected 30 min before BA administration (20 mg/kg) for BA + 3MA and BA+ CC groups. The dose and time of BA administration were used according to the previous study 35. All drugs were injected for 3 days after SCI. All animals were sacrificed by overdose of pentobarbital sodium and histological samples were harvested on day 3, with the exception of 20 mice designated for Hematoxylin and Eosin (H&E) and Masson staining and to assess locomotion recovery.

### Functional behavior assessment

The Basso mouse scale (BMS) locomotion scale and the footprint test were administered at 0, 1, 7, 14, 21, and 28 days after SCI to evaluate functional recovery 36. The BMS score ranges from 0 to 9 points, with 0 indicating complete paralysis and indicating normal motor functions. Footprint analysis was performed by first immersing the hind paws in black dye, and then allowing the animals to walk for 5 min as described previously 37. Outcome measures were measured by two independent examiners blinded to experimental conditions.

### Tissue slides preparation for HE and Masson staining

On postoperative day 14, mice in the Sham, SCI, BA, and BA +3MA groups were re-anesthetized with 2% (w/v) pentobarbital sodium and perfused with normal saline, followed by addition of 4% (w/v) paraformaldehyde in phosphate-buffered saline. Then, we separated the whole segments (10mm in length, epicenter in middle), then post-fixed them for 24 h in 4% (w/v) paraformaldehyde. Subsequently, each specimen was prepared for longitudinal paraffin sections after being embedded in paraffin. Using a microtome, 4 μm sections were cut and mounted on poly-L-lysine-coated slides for histopathological examination by HE staining as previously described 1, 38. For Masson staining, we used 10% potassium dichromate and 10% trichloroacetic acid to mordant longitudinal sections and we used hematoxylin to stain nuclei. Then, using hydrochloric acid and ethanol, slides were differentiated, returned to blue with weak ammonia, and stained with the Masson solution; this staining protocol was previously described 39. Finally, a light microscope (Olympus, Tokyo, Japan) was used to photograph images.

### Western blot analysis

After mice had been euthanized on day 3 following SCI, the spinal cord segments in mice (1.5 cm; containing the injury epicentre) were dissected and stored for Western blot analyses at -80 °C. A part of the samples was processed by extracting proteins with a lysis buffer. Other samples were processed by extracting cytoplasmic protein and nuclear protein with NE-PER™ Nuclear and Cytoplasmic Extraction Reagents. We used protein extraction reagents to purify total proteins from spinal cord specimens and used the BCA assay to measure the proteins. 12% (w/v) gel electrophoresis was used to separate an equal amount of 60 μg protein, which were then transferred to polyvinylidene fluoride membranes (Roche Applied Science, Indianapolis, IN, USA). After blocking for 2 h at room temperature with 5% (w/v) non-fat milk, the membranes were then incubated overnight at 4 °C with the subsequent primary antibodies: mTOR (1:1,000), p-mTOR (1:1,000), AMPK (1:1,000), p-AMPK (1:1,000), NLRP3 (1:1,000), TFEB (1:1,000), IL-1β (1:1,000), IL-18 (1:1,000), GSDMD (1:1,000), Nix (1:1,000), ASC (1:1,000), Parkin (1:1,000), Bnip3 (1:1000), Beclin1 (1:1,000), Caspase 1 (1:1,000), LC3 (1:500), p62 (1:1,000), CTSD (1:1,000), Vps34 (1:1,000), and GAPDH (1:1,000). Finally, membranes were incubated for 2 h at room temperature with HRP-conjugated IgG secondary antibody (1:5,000). ECL Plus Reagent Kit was used to visualize band signals, and Image Lab 3.0 software (Bio-Rad, Hercules, CA, USA) was used to quantify band intensities.

### Enzyme-linked immunosorbent assay (ELISA)

We homogenized, frozen and thawed spinal cord specimens repeatedly in liquid nitrogen. Then, we centrifuged (at 10,000 g) the homogenate at 4 °C for 10 minutes, and collected the supernatant for ELISA. ELISA kits were used to evaluate the levels of AOPP, 8-OHdG, and MDA in spinal cord specimens according to manufacturer's protocols (Boyun Biotech, Shanghai, China). Finally, the quantifications of AOPP, 8-OHdG, and MDA were performed using a microplate reader at 550 nm with a correction wavelength of 450 nm.

### Immunofluorescence staining

Immunofluorescence staining for tissue sides from the rostral spinal cord segments (1mm in length, 4mm far from epicenter) were performed as previously described 32. We deparaffinized, rehydrated, washed, and then treated sections at 95 °C for 20 min with 10.2mM sodium citrate buffer. Then, 0.1% (v/v) PBS-Triton X-100 was used to permeabilize the sections (10 minutes), and used 10% (v/v) bovine serum albumin in PBS was used for blocking (1 hour). After that, slides were incubated overnight at 4°C with antibodies against p62 (1:200)/NeuN (1:400), LC3 (1:200)/NeuN (1:400), GSDMD (1:150)/NeuN (1:400), Caspase-1/NeuN, and SYN (1:200)/NeuN (1:400), Nix (1:150)/NeuN (1:400), and MAP2 (1:200). Then, we washed the sections for 10 minutes at room temperature three times and incubated the sections at room temperature for 1 hour with FITC-conjugated secondary antibody. Finally, we captured and assessed images taken by a fluorescence microscope (Olympus, Tokyo, Japan) in 6 randomly selected fields from 3 random sections of each sample.

### Statistical analyses

All statistical analyses were performed on SPSS 19.0 (SPSS, Chicago, IL). All data are presented as mean ± Standard Error of Mean (SEM). Comparisons between two independent groups were performed using independent-sample t-test. Comparisons among more than three groups was performed using one-way ANOVA with LSD (equal variances assumed). *P* < 0.05 was considered statistically significant.

## Results

### BA promotes functional recovery after SCI

**Figure 1:**
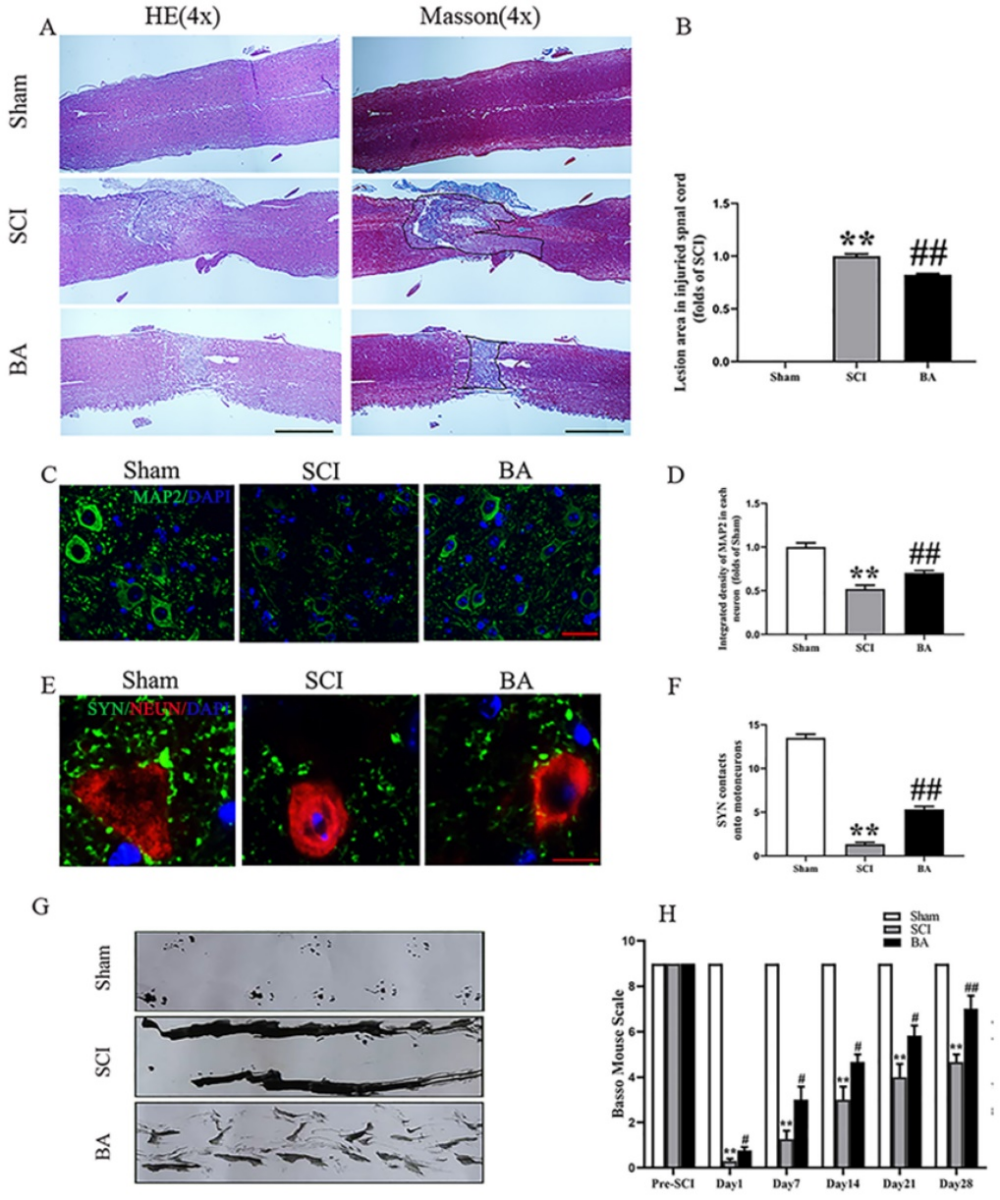
** BA promotes functional recovery after SCI. (A)** Longitudinal spinal cord sections from the indicated groups at day 14 were analyzed by HE staining and Masson staining (scale bar = 1000 µm). **(B)** Quantitative analysis of Masson positive lesions in the spinal cords of each group. **(C)** Images (30×) of the spinal cord sections in each group stained with antibodies against MAP2 (scale bar = 25 µm). **(D)** The optical density of MAP2 in the injured spinal cord at day 28. **(E)** Images (150×) of spinal cord sections below the injury (T11-T12) stained at day 28 with antibodies against SYN/NeuN (scale bar = 5 µm) **(F)** Corresponding quantification of the number of synapses contacting motor neurons. **(G)** Photos of mice footprints at day 28 after SCI. **(H)** Basso mouse scale (BMS) for the indicated groups and time points. The values are expressed as the means ± SEM, n=5 per group. ^**^p< 0.01, vs. Sham group. ^#^*p*< 0.05 and ^##^*p*< 0.01, vs. SCI group.

Compared to sham control, the SCI group showed an expanded area of glial scar (*p*<0.001), down-regulated MAP2 (*p* < 0.001), and less SYN-positive synapses onto ventral motor neurons (*p* < 0.001). With BA treatment, animals had less glial scar, higher neuronal MAP2 expression, and more SYN-positive synapses onto ventral motor neurons compared with the untreated SCI group (*p* < 0.001, *p* < 0.001, *p* = 0.006, respectively; **Fig. 1A-F**). Moreover, footprint analysis showed that the BA group fared better than SCI group for functional recovery at 28 days after injury (**Fig. 1G**). For the Sham group, the BMS scores were notably higher compared to the SCI group at days 1, 7, 14, 21 and 28 post-procedure (*p*<0.001 for all). Similarly, the BA group also had higher BMS scores at days 1, 7, 14, 21 and 28 post-procedure compared with the SCI group (*p* = 0.018, *p* = 0.021,* p* = 0.022, *p* = 0.021,* p* = 0.005, respectively; **Fig. 1H**). Together, these results demonstrate that BA promotes functional recovery following SCI.

### BA attenuates pyroptosis after SCI

**Figure 2:**
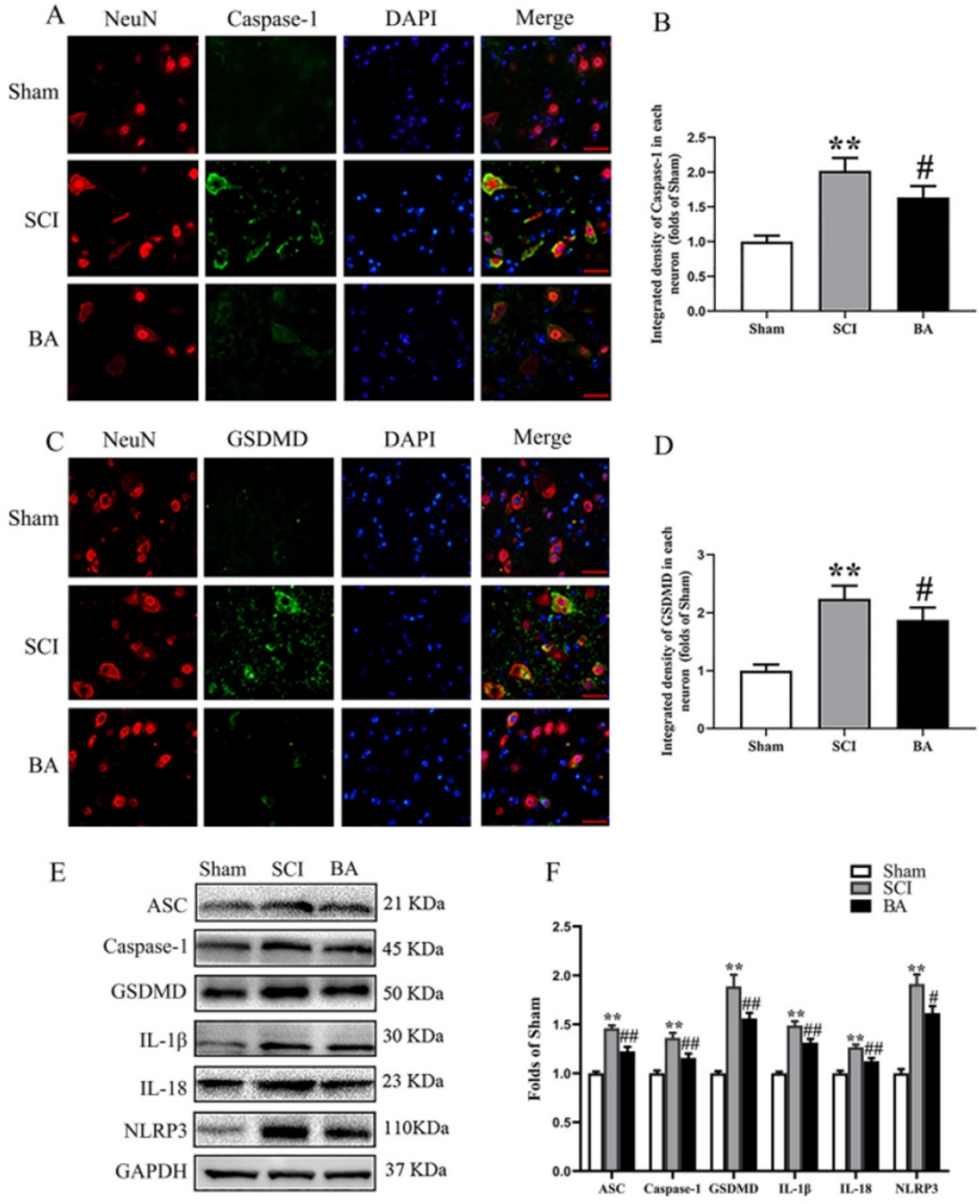
** BA attenuates pyroptosis after SCI. (A)** Immunofluorescence staining for Caspase-1 and NeuN co-localization in the spinal cords of the Sham, SCI, and BA groups (scale bar = 25 µm)** (B)** The quantitative mean optical density of the Caspase-1 in motor neurons of spinal cord lesion. **(C)** Immunofluorescence staining for GSDMD and NeuN co-localization in the spinal cords of the Sham, SCI, and BA groups (scale bar = 25 µm)** (D)** The quantitative mean optical density of the GSDMD in motor neurons of spinal cord lesion.** (E)**Western blotting for ASC, Caspase-1, GSDMD, IL-1β, IL-18 and NLRP3 expression levels in the Sham, SCI, and BA groups. The gels were run under the same experimental conditions, and the cropped blots are shown here. **(F)** The optical density values of the ASC, Caspase-1, GSDMD, IL-1β, IL-18 and NLRP3 expression levels were quantified and analyzed in each group. The values are expressed as the means ± SEM, n=5 per group. **p< 0.01, vs. Sham group. ^#^*p*< 0.05 and ^##^*p*< 0.01, vs. SCI group.

ASC, GSDMD, Caspase-1, NLRP3, IL-1β, and IL-18 were assessed in the spinal cord after SCI to assess pyroptotic activity in Sham, SCI and BA groups. As shown in **Fig. 2A-D**, immunofluorescence staining showed that Caspase-1 and GSDMD density in neurons were significantly increased in the spinal cord lesions in the SCI group relative to the Sham group (*p* < 0.001 for both), while BA decreased the densities of Caspase-1 and GSDMD compared with the SCI group (*p* = 0.016,* p* = 0.013, respectively). Western blots for ASC, GSDMD, Caspase-1, NLRP3, IL-1β, and IL-18 expressions levels were also assessed (**Fig. 2E**). Results demonstrated that the OD values for ASC, Caspase-1, GSDMD, IL-1β, IL-18 and NLRP3 were higher in the SCI group compared with the Sham group (*p* < 0.001 for all), and that BA decreases in the OD values for these markers relative to the SCI group (*p* < 0.001, *p* = 0.009, *p* = 0.003, *p* = 0.004, *P* = 0.004, *P* = 0.013, respectively; **Fig. 2F**). These results suggest that BA reduces pyroptosis-related markers, suggestive of an inhibitory effect on pyroptosis following SCI.

### BA enhances autophagy after SCI

**Figure 3:**
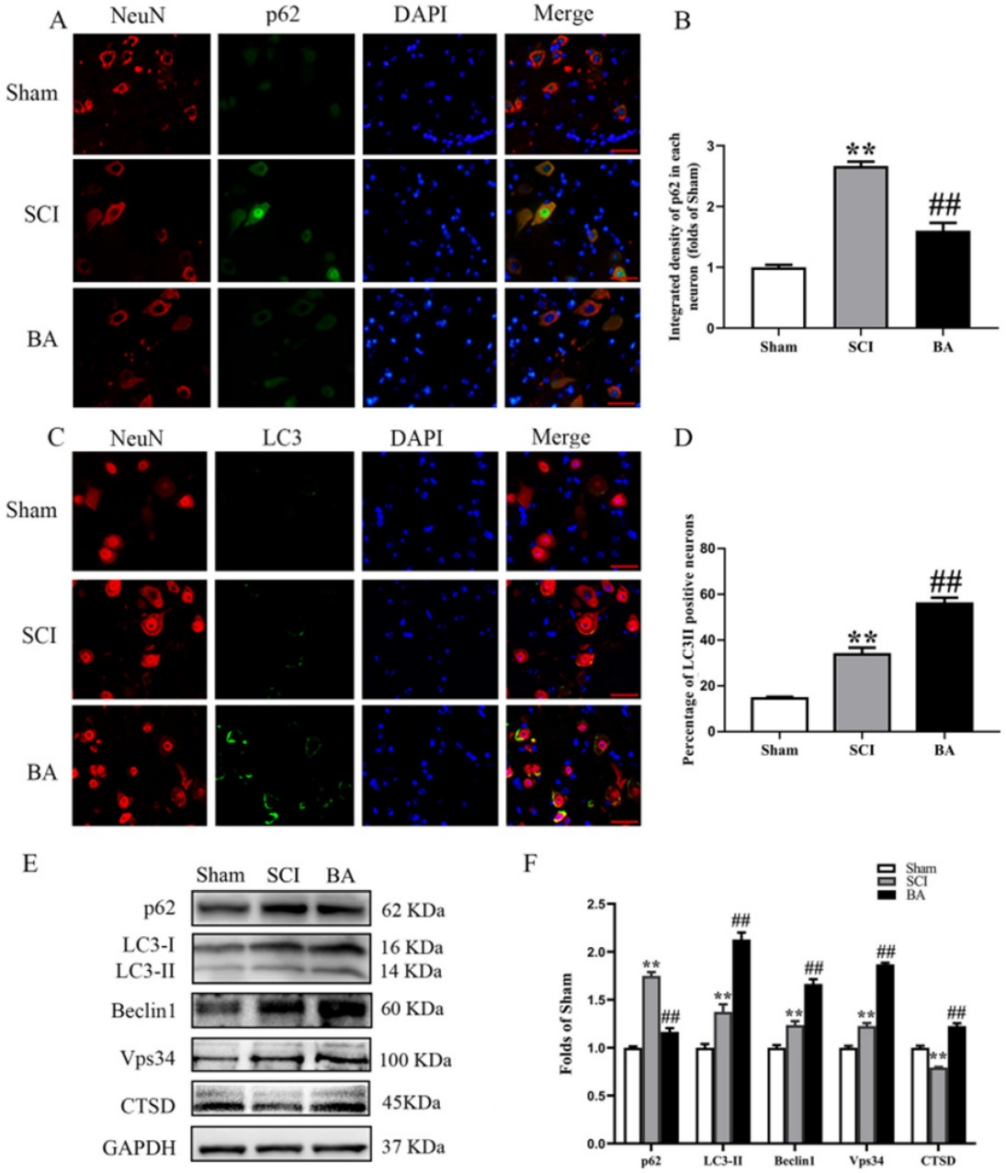
** BA enhances autophagy after SCI. (A)** Immunofluorescence staining for p62 and NeuN co-localization at the spinal cord lesion after SCI (scale bar = 25 µm).** (B)** The quantitative mean optical density of the p62 in motor neurons of spinal cord lesion in each group. **(C)** Immunofluorescence staining for LC3 and NeuN co-localization at the spinal cord lesion after SCI (scale bar = 25 µm). **(D)**The percentage of the LC3II positive neurons in motor neurons of spinal cord lesion in each group.** (E)** Western blotting for p62, LC3II, Beclin1, Vps34, and CTSD expression levels in the Sham, SCI and BA groups. The gels were run under the same experimental conditions, and the cropped blots are shown here. **(F)** The optical density values of the p62, LC3II, Beclin1, Vps34, and CTSD expression levels were quantified and analyzed in each group. The values are expressed as the means ± SEM, n=5 per group. **p< 0.01, vs. Sham group. ^##^*p*< 0.01, vs. SCI group.

To assess autophagic activity in the spinal cord lesion after SCI, we measured protein levels of autophagosomal markers (LC3II, Beclin1 and Vps34), an autolysosome-related marker (CTSD), and an autophagic substrate protein (p62). As shown in **Fig. 3A**, immunofluorescence staining revealed p62 levels in the neurons at the lesion; a green label was used to mark p62 was labeled green, a red label for neurons, and a blue label for nuclei. Quantitative analyses showed that after SCI, the percentage of p62-positive neurons significantly increased (*p* < 0.001); however, the BA group had a lower percentage of p62-positive neurons compared with the SCI group (*p* < 0.001; **Fig. 3B**). To assess LC3II levels, a green label was applied for LC3II, a red label for neurons (NeuN), and blue label for nuclei (DAPI). As shown in **Fig. 3C**, the spinal cord exhibited higher percentage of LC3Ⅱ positive neurons in the SCI group than in the Sham group (*p* < 0.001); BA treatment further increased LC3Ⅱ positive neurons compared with the SCI group (*p* < 0.001; **Fig. 3D**). The amount of p62, LC3II, Beclin1, Vps34, and CTSD proteins were measured by Western blot (**Fig. 3E**). Results showed that the OD of p62, LC3II, Beclin1, Vps34, were higher in the SCI group than in the Sham group (*p* < 0.001, *p* = 0.001, *p* = 0.001, *p* < 0.001, respectively), with lower OD values for CTSD in the SCI group (*p* < 0.001). BA enhanced the level of LC3II, Beclin1, Vps34, and CTSD as well as decreased level of p62 in the BA group compared with the SCI group (*p*<0.001 for all; **Fig. 3F**). These results recapitulate the known phenomenon that following SCI, autophagy substrates accumulate despite an upregulation autophagosome- and autolysosome-related markers. These results also demonstrate that BA is not only able to increase autophagosome- and autolysosome-related markers, it also alleviates autophagy substrate burden, likely due to an inducing an overall increase in autophagic activity following SCI.

### Inhibition of autophagy reverses the effects of BA on pyroptosis after SCI

**Figure 4:**
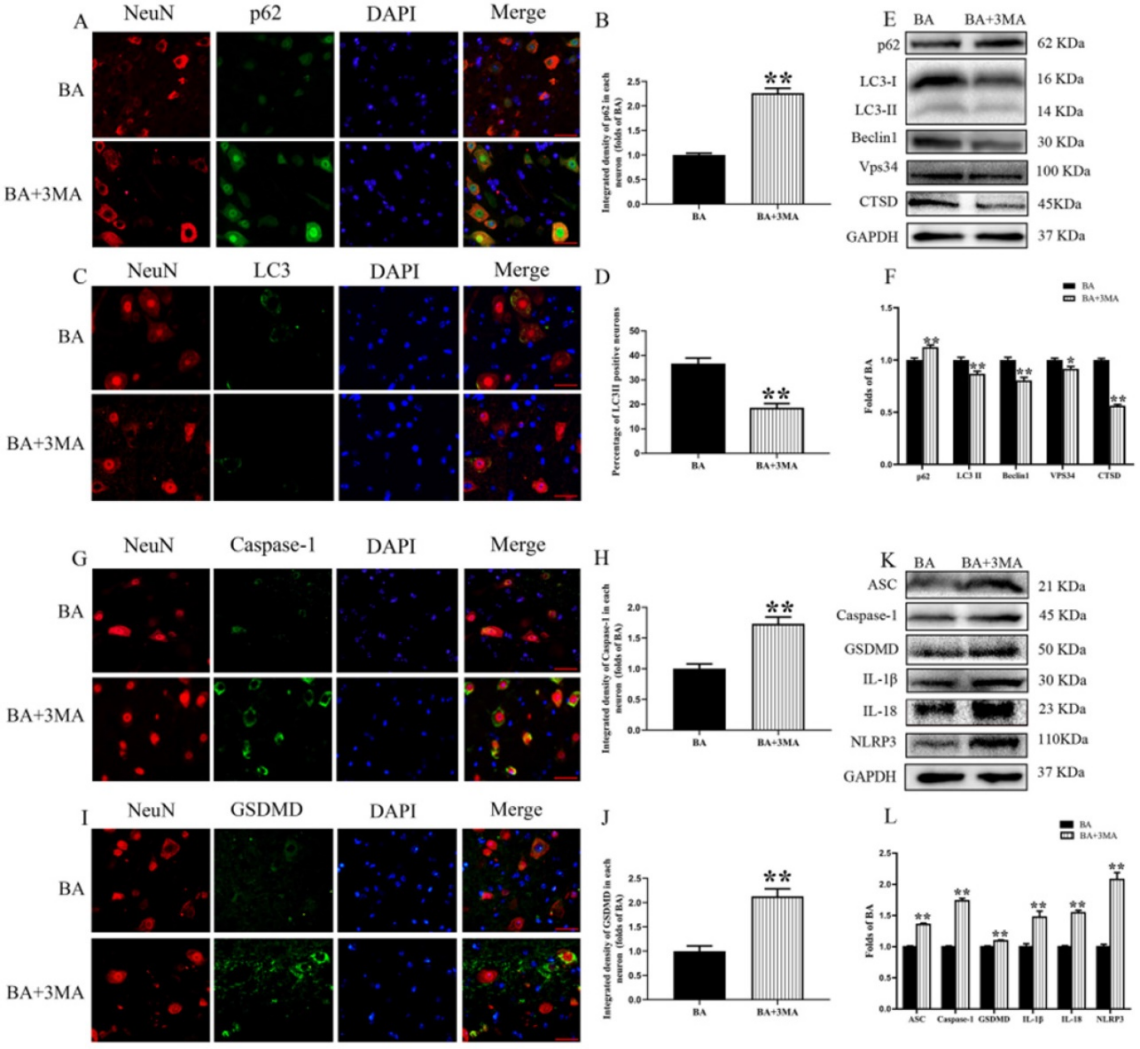
** Inhibition of autophagy reverses the effects of BA on pyroptosis after SCI. (A)** Immunofluorescence staining for p62 and NeuN co-localization at the spinal cord lesion after SCI (scale bar = 25 µm). **(B)** The quantitative mean optical density of the p62 in motor neurons of spinal cord lesion in each group. **(C)** Immunofluorescence staining for LC3II and NeuN co-localization at the spinal cord lesion after SCI (scale bar = 25 µm). **(D)** The quantitative mean number of the LC3II positive neurons in motor neurons of spinal cord lesion in each group. **(E)** Western blotting for the p62, LC3II, Beclin1, Vps34, and CTSD expression levels in the BA and BA+3MA groups. The gels were run under the same experimental conditions, and the cropped blots are shown here. **(F)** The optical density values of the p62, LC3II, Beclin1, Vps34, and CTSD expression levels were quantified and analyzed in each group.** (G)** Immunofluorescence staining for Caspase-1 and NeuN co-localization in the spinal cords of the BA and BA+3MA groups (scale bar = 25 µm)** (H)** The quantitative mean optical density of the Caspase-1 in motor neurons of spinal cord lesion. **(I)** Immunofluorescence staining for GSDMD and NeuN co-localization in the spinal cords of the BA and BA+3MA groups (scale bar = 25 µm) **(J)** The quantitative mean optical density of the GSDMD in motor neurons of spinal cord lesion. **(K)** Western blotting for the ASC, Caspase-1, GSDMD, IL-1β, IL-18 and NLRP3 expression levels in the BA and BA+3MA groups. The gels were run under the same experimental conditions, and the cropped blots are shown here. **(L)** The optical density values of the ASC, Caspase-1, GSDMD, IL-1β, IL-18 and NLRP3 expression levels were quantified and analyzed in each group. The values are expressed as the means ± SEM, n=5 per group. ^*^p< 0.05 and ^**^p< 0.01, vs. BA group.

3MA, an autophagy inhibitor, was co-administered with BA to assess whether BA's beneficial effects on outcomes following SCI is due to autophagy activation. Immunofluorescence and neuron co-localization analyses revealed increased p62 density and decreased LC3II signals BA+3MA group compared with the BA group (*p* < 0.001, *p* < 0.001, respectively; **Fig. 4A-D**). The expression levels of p62, LC3II, Beclin1, Vps34, and CTSD were detected by Western blot (**Fig. 4E**). Results showed that the OD values for LC3II, Beclin1, Vps34, and CTSD were lower in the BA+3MA group than in the BA group (*p* = 0.004, *p* = 0.001,* p* = 0.017, *p* < 0.001, respectively), with a higher OD value for p62 in the BA+3MA group (*p* = 0.001; **Fig. 4F**). These results demonstrate that 3MA was effective in inhibiting autophagy when co-administered with BA. Next, pyroptotic activity was assessed in BA-treated animals via immunofluorescence staining and Western blotting after co-administration of 3MA. Immunofluorescence showed that Caspase-1 and GSDMD densities in neurons were higher in the BA+3MA group than in the BA group (*p* < 0.001 for both; **Fig. 4G-J**). The expression levels of ASC, GSDMD, Caspase-1, NLRP3, IL-1β, and IL-18 were also measured by Western blot (**Fig. 4K**). Results revealed that the OD values for ASC, Caspase-1, GSDMD, IL-1β, IL-18 and NLRP3 were higher in the BA+3MA group than in the BA group (*p* < 0.001, *p* < 0.001,* p* = 0.001, *p* =0.002, *p* =0.002, *p* < 0.001 respectively; **Fig. 4L**). These results show that co-administration of 3MA with BA leads to a reduction in BA's effect on reducing pyroptosis, suggesting that the autophagy-enhancing effects of BA may underly the mechanism by which it inhibits pyroptosis.

### Autophagy inhibition reverts the effects of BA on functional recovery after SCI

**Figure 5:**
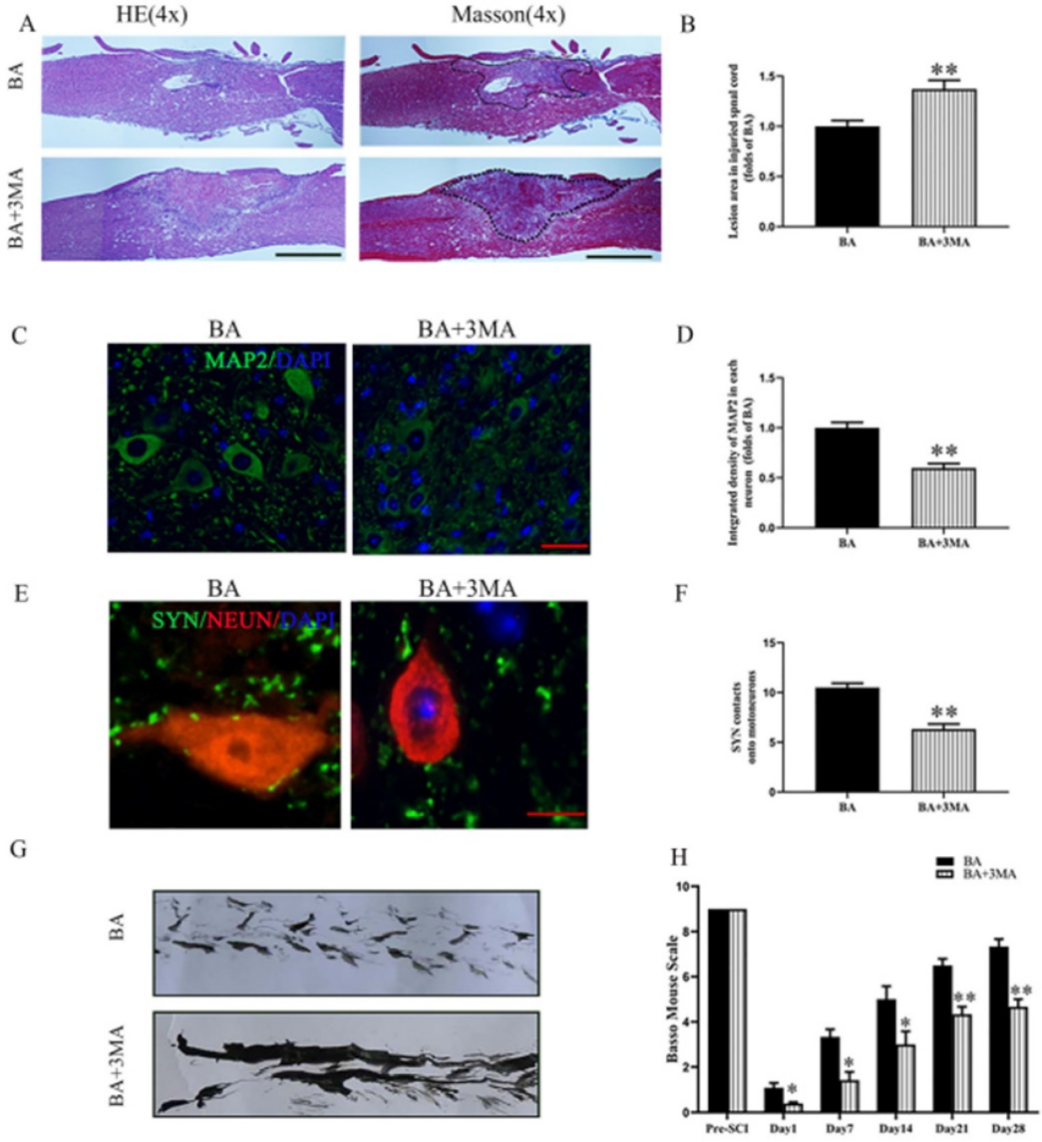
** Inhibition of autophagy reverses the effects of BA on functional recovery after SCI. (A)** Longitudinal spinal cord sections from the indicated groups at day 14 were analyzed by HE staining and Masson staining (scale bar=1000 µm). **(B)** Quantitative analysis of Masson positive lesions in the spinal cords of each group. **(C)** Images (30×) of the spinal cord sections in each group stained with antibodies against MAP2 (scale bar = 25 µm). **(D)** The optical density of MAP2 in the injured spinal cord at day 28. **(E)** Images (150×) of spinal cord sections below the injury (T11-T12) stained at day 28 with antibodies against SYN/NeuN (scale bar = 5 µm).** (F)** Corresponding quantification of the number of synapses contacting motor neurons. **(G)** Photos of mice footprints at day 28 after SCI. **(H)** Basso mouse scale (BMS) for the indicated groups and time points. The values are expressed as the means ± SEM, n=5 per group. ^*^p< 0.05 and ^**^p< 0.01, vs. BA group.

Compared to the BA group, the BA+3MA group showed an increased area of glial scar (*p* = 0.005, **Fig. 5A-B**), decreased MAP2 levels (*p* < 0.001, **Fig. 5C-D**) and lower number of SYN-positive synapses onto ventral motor neurons following SCI (*p* < 0.001, **Fig. 5E-F**). At day 28 after injury, the BA group showed a significant restoration of hind legs movement with coordinated crawling, whereas the BA+3MA group was still dragging their hind legs (**Fig. 5G**). In the BA+3MA group, the BMS scores were significantly lower than those in the BA group after SCI at days 1, 7, 14, 21 and 28 (*p* = 0.032, *p* = 0.017, *p* = 0.025, *p* = 0.008, *p* = 0.005, respectively; **Fig. 5H**). These results suggest that BA's autophagy enhancing effects may be responsible for improved outcomes with BA treatment following SCI.

### BA enhances mitophagy and reduces ROS accumulation after SCI

**Figure 6:**
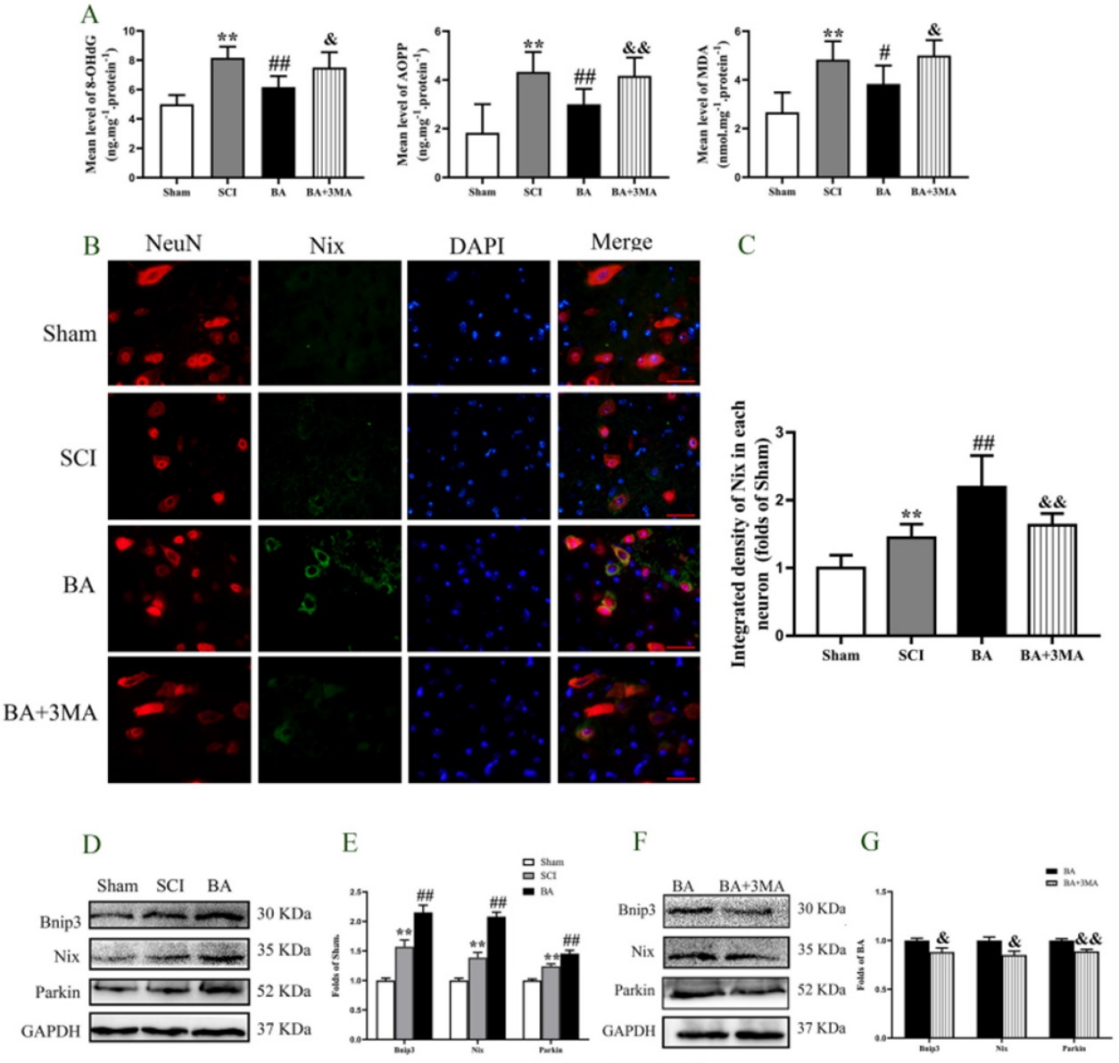
** BA attenuates mitophagy and reduces ROS accumulation after SCI. (A)** ELISA of 8-OHdG, AOPP, and MDA in spinal cord lesions from Sham, SCI, BA and BA+3MA groups as indicated. **(B)** Immunofluorescence staining for Nix and NeuN co-localization in the spinal cords of the Sham, SCI, BA and BA+3MA groups (scale bar = 25 µm). **(C)** The quantitative mean optical density of the Nix in motor neurons of spinal cord lesion in each group. **(D)** Western blotting for Bnip3, Nix and Parkin expression levels in the Sham, SCI and BA groups. The gels were run under the same experimental conditions, and the cropped blots are shown here.** (E)** The optical density values of the Bnip3, Nix and Parkin expression levels were quantified and analyzed in the three groups. **(F)** Western blotting for Bnip3, Nix and Parkin expression levels in the BA and BA+3MA groups. The gels were run under the same experimental conditions, and the cropped blots are shown here. **(G)** The optical density values of the Bnip3, Nix and Parkin expression levels were quantified and analyzed in the both groups. The values are expressed as the means ± SEM, n=5 per group. *p< 0.05 and **p< 0.01, vs. Sham group. ^#^*p*< 0.05 and ^##^*p*< 0.01, vs. SCI group.^ &^*p*< 0.05 and ^&&^*p*< 0.01, vs. BA group.

ROS oxidation products -AOPP, 8-OHdG, and MDA - were measured by ELISA to evaluate changes in ROS levels after SCI. The levels of AOPP, 8-OHdG, and MDA were all higher in the SCI group compared with the Sham group (*P* < 0.001 for all). BA treatment decreased the levels of AOPP, 8-OHdG, and MDA (*p* < 0.001, *p* < 0.001,* p* = 0.041, respectively), and the levels of AOPP, 8-OHdG, and MDA were all higher in the BA+3MA group compared with the BA group (*p* = 0.004,* p* = 0.010, *p* = 0.013, respectively, **Fig. 6A**). Biomarkers Bnip3, Nix and Parkin assessed to measure mitophagy in the Sham, SCI, BA and BA+3MA groups. Immunofluorescence showed that there were significantly more Nix-positive neurons in the SCI group relative to the Sham group (*p* = 0.026), and more Nix-positive neurons were observed in the BA group compared with the SCI group (*p* =0.001; **Fig. 6B, C**). Furthermore, compared to BA group, there were less Nix-positive neurons in the BA+3MA group (*p* < 0.001; **Fig. 6B, C**). The expression levels of Bnip3, Nix and Parkin were also measured by Western blot (**Fig. 6D**). Results showed that Bnip3, Nix and Parkin levels were significantly higher in the SCI group compared to the Sham group (*p* = 0.001, *p* = 0.002,* p* = 0.001, respectively), and these levels were even higher in the BA group compared with the SCI group (*p* = 0.001, *p* < 0.001,* p* = 0.003, respectively; **Fig. 6E**). Finally, compared to BA group, the expression levels of Bnip3, Nix and Parkin in the BA+3MA group were all lower (*p* = 0.041, *p* = 0.018,* p* = 0.002, respectively; **Fig. 6F, G**). Together, these results indicate that BA reduces ROS accumulation and augments mitophagy in SCI, and that these effects are likely due to its autophagy enhancing effects.

### BA activates autophagy and mitophagy via enhancing AMPK-mTOR -TFEB activity

**Figure 7:**
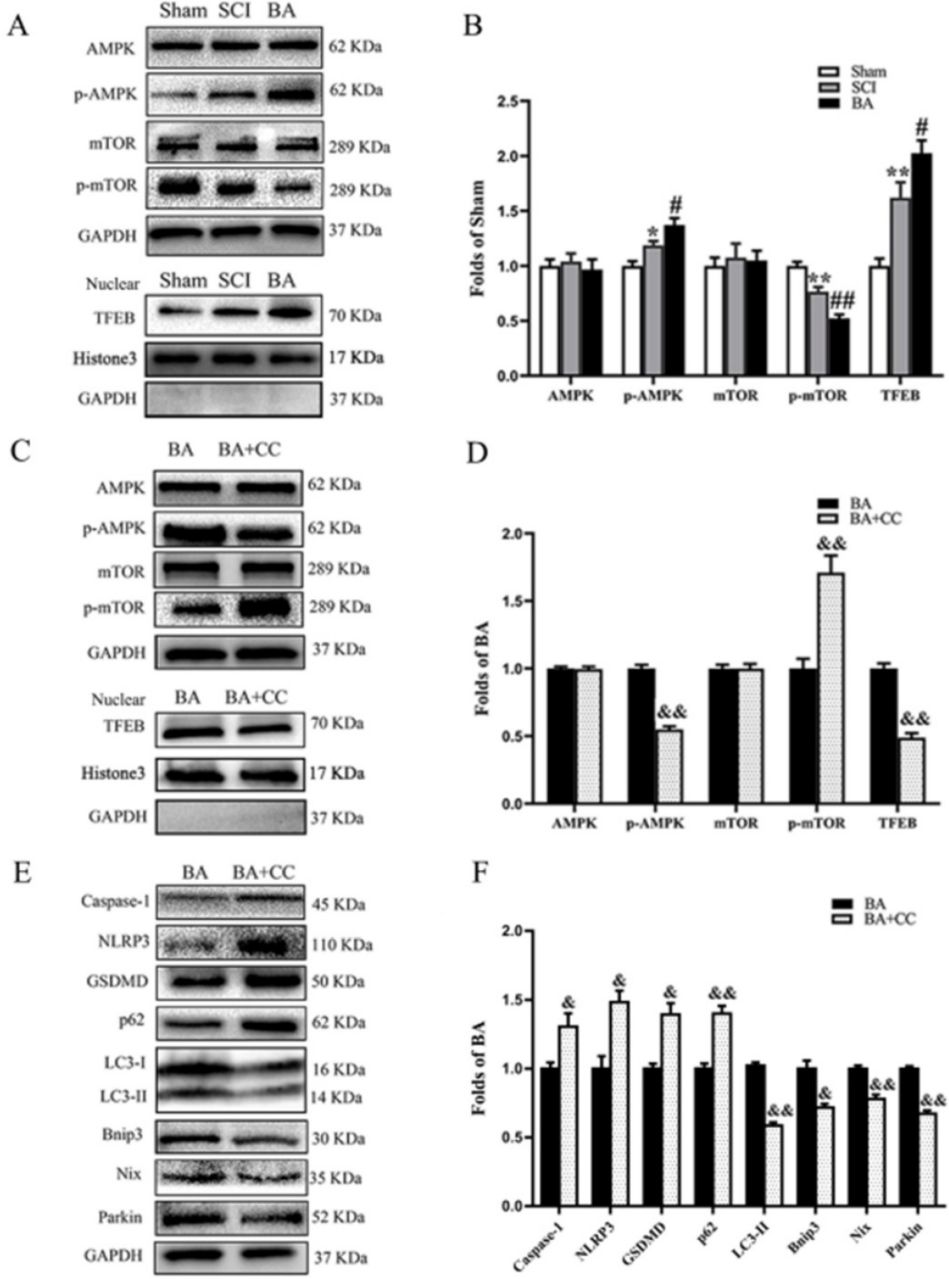
** BA activates autophagy and mitophagy via enhancing AMPK-mTOR -TFEB activity. (A)** Western blotting for AMPK, p-AMPK, mTOR and p-mTOR expression levels, and TFEB nuclear translocation in the Sham, SCI and BA groups. The gels were run under the same experimental conditions, and the cropped blots are shown here.** (B)** The optical density values of the AMPK, p-AMPK, mTOR, p-mTOR, normalized to the loading control GAPDH; Densitometric analysis of TFEB, normalized to the loading control H3. **(C)** Western blotting for AMPK, p-AMPK, mTOR and p-mTOR expression levels, and TFEB nuclear translocation in the BA and BA+3MA groups. The gels were run under the same experimental conditions, and the cropped blots are shown here. **(D)** The optical density values of the AMPK, p-AMPK, mTOR, p-mTOR, normalized to the loading control GAPDH; Densitometric analysis of TFEB, normalized to the loading control H3. **(E)** Western blotting for Caspase-1, NLRP3, GSDMD, p62, LC3II, Bnip3, Nix and Parkin expression levels in the BA and BA+CC groups. The gels were run under the same experimental conditions, and the cropped blots are shown here. **(F)** The optical density values of the Caspase-1, NLRP3, GSDMD, p62, LC3II, Bnip3, Nix and Parkin expression levels, normalized to the loading control GAPDH. The values are expressed as the means ± SEM, n=5 per group. *p< 0.05 and **p< 0.01, vs. Sham group. ^#^*p*< 0.05 and ^##^*p*< 0.01, vs. SCI group.^ &^*p*< 0.05 and ^&&^*p*< 0.01, vs. BA group.

We sought to investigate the mechanism underlying how BA modulates pyroptosis, autophagy and mitophagy. We assessed whether TFEB, a known activator of autophagy, is involved. Western blot analysis revealed that p-AMPK expression and TFEB nuclear translocation levels were higher in the SCI group compared with the Sham group (*p* = 0.038, *p* = 0.008, respectively), while p-mTOR was lower (*p* = 0.005). BA increased the levels of p-AMPK expression and TFEB nuclear translocation and decreased p-mTOR level (*p* = 0.039, *p* = 0.044,* p* = 0.004, respectively, **Fig. 7A, B**). Next, we explored the effects of compound C (CC), an AMPK blocker, on the effects of BA. Here, p-AMPK and TFEB nuclear translocation levels were lower in the BA+CC group compared with the BA group (*p* <0.001,* p* = 0.001, respectively), while p-mTOR levels in the BA+CC group was higher (*p* = 0.008, **Fig. 7C, D**). We also evaluated whether the AMPK-mTOR -TFEB axis is also involved in the mechanism by which BA modulates pyroptosis, autophagy, and mitophagy related proteins. Our results demonstrated that Caspase-1, NLRP3 and GSDMD levels were higher in the BA+CC group when compared to the BA group (*p* = 0.046, *p* = 0.018,* p* = 0.012, respectively); p62 levels were higher in the BA+CC group when compared to the BA group (*p* = 0.004), with a lower level of LC3II (*P* = 0.009). Finally, Bnip3, Nix and Parkin were lower in the BA+CC group when compared to the BA group (*p* = 0.012, *p* = 0.005, *p* <0.001, respectively, **Fig. 7E, F**). Together, these findings suggest that BA activates autophagy, inhibits pyroptosis, and augments mitophagy via the AMPK-mTOR-TFEB pathway.

## Discussion

Betulinic acid (BA), a natural pentacyclic triterpenoid, has gained considerable attention in recent years for its strong biological and medicinal properties 40. Increasing evidence suggest that BA plays a substantial role in the treatment of various nervous system diseases such Alzheimer's disease 41 peripheral neuropathies 42. Spinal cord injury is a devastating and common disease that inflicts substantial physiological, emotional, and economic damage to patients, their families and societies worldwide. In this study, we present novel preclinical evidence that BA may contribute functional outcomes following SCI. Mechanistically, our results demonstrate that BA's therapeutic effect was likely due to autophagy augmentation via the AMPK-mTOR-TFEB pathway, subsequently inducing mitophagy, suppressing ROS accumulation, and inhibiting pyroptosis.

Autophagy, a lysosomal-dependent degradation pathway for intracellular proteins and organelles, plays an important role in human health and disease 43. In the context of SCI, autophagy has shown to play an important role, albeit a complicated one. On one hand, melatonin-induced increase in autophagy has been shown to promote locomotor recovery in SCI 44, but on the other hand, induction of autophagy may lead to neuronal cell death 45. Despite lingering controversy, accumulating evidence seems to suggest that autophagy is primarily beneficial in the context of SCI 46, 47. Our previous work revealed that BA was an effective activator of autophagy 35, and thus, we hypothesized that BA may be effective in improving outcomes following SCI. In the present study, not only did we find that BA improved outcomes following SCI, WB and immunofluorescence staining results also suggested that these beneficial effects are largely due to up-regulation of autophagy, and that 3MA reverses these benefits.

Pyroptosis is another cellular pathway that has also been implicated in a variety of pathologies such as colitis, myocardial dysfunction, and neuronal damage 48-50. In brief, pyroptosis is a form of inflammatory programmed cell death characterized by inflammasome activation, membrane pore-formation, swelling, rupture, and ultimate dissolution and release of intracellular contents 51. In the canonical inflammasome pathway, NLRP3 inflammasomes are activated to recruit ASC to form ASC focus 51, 52. Then, ASC focus activated Caspase-1, which cleaves pro-IL-18/1β and gasdermin D (GSDMD) to release their mature forms 51. IL-18/1β and GSDMD are then released to the extracellular domain and subsequently trigger a robust inflammatory response 53. In this study, we hypothesized that BA's augmentation of autophagy may be able to suppress pyroptosis in SCI. Our Western blot and immunofluorescence results showed that BA was able to significantly depressed pyroptosis-associated markers such as ASC, NLRP3, GSDMD, Caspase-1, IL-1β and IL-1B, suggesting that BA may be an effective inhibitor of pyroptosis in SCI. Furthermore, we found that 3MA co-administration abates these pyroptosis inhibiting effects, suggesting that BA likely suppresses pyroptosis via enhancing autophagy.

Increasing evidence demonstrates that the secondary injury stage after SCI is closely related to mitochondrial injury and excessive ROS generation 54. Damaged mitochondria leads to a massive accumulation of ROS, which can induce NLRP3 inflammasome activation and subsequently trigger caspase-1-dependent pyroptosis 55, 56. Mitophagy, a selective autophagic degradation of damaged mitochondria 57, can reduce ROS accumulation 58, 59, and may play a central role in curtailing pyroptosis following SCI. To initiate mitophagy, autophagy receptors p62 and optineurin (OPTN) have been shown to bind ubiquitin chains on damaged mitochondria 60. Although some studies suggest a contribution of autophagy to mitophagy, its contribution is largely unknown 61, 62. To explore this postulation, we investigated whether BA-mediated autophagy enhancement promotes mitophagy, and whether mitophagy underlies the mechanism by which BA inhibits pyroptosis following SCI. Our results showed that BA significantly elevated the levels of Bnip3, Nix and Parkin following SCI, suggesting an upregulation of mitophagy. Furthermore, our findings demonstrate that BA decreased ROS oxidation products, including 8-OHdG, AOPP and MDA. These changes were abated by co-administration of 3MA, suggesting that mitophagy activation and ROS reduction occurred downstream of autophagy enhancement. It was suggested that in SCI, ROS is likely responsible for pyroptosis activation, and that autophagy-induced mitophagy activation and ROS reduction may underly the mechanism by which BA suppresses pyroptosis.

In order to further to elucidate the underlying mechanism of how BA promotes autophagy in SCI, we also examined upstream mechanisms of autophagy. Transcription factor EB (TFEB) is a central regulator of autophagy 63, 64. Past studies have shown that TFEB activation occurs in response to a variety of cellular stress 65, which impacts the ATP to ADP ratio, triggering the phosphorylation of AMPK. Subsequently, AMPK regulates cell metabolism 66, inhibiting mTOR (a master regulator of cell growth and metabolism) in the cytoplasm to allow nuclear translocation of TFEB 67. In our present work, we showed that BA increases the expression of TFEB. Furthermore, our results revealed that BA leads to increased phosphorylation of AMPK and inhibited the phosphorylation of mTOR. Finally, using compound C (CC), an AMPK blocker, we showed that inhibition of AMPK-mTOR-TFEB signaling pathway led to diminished effects of BA on autophagy, and mitophagy, and pyroptosis. The activated AMPK-mTOR pathway also promotes release of Ca^2+^ through TRPML1 channels, which activate calcineurin 68. Calcineurin is a calcium ion-dependent phosphatase, and activated calcineurin promotes the nuclear translocation of TFEB 63. Thus, BA may promote TFEB-induced autophagy via APMK-TRPML1-calcineurin pathway following SCI, which needs to be verified in the further study.

Naturally, there are several limitations in our study that need to be further investigated. For example, previous studies revealed that AMPK-SPK2-CARM1 signaling pathway is another important pathway regulating TFEB in the nucleus 69, 70, and future investigations should explore whether BA also acts through AMPK -SPK2-CARM1-TFEB signaling pathway in SCI. 28-days slices were commonly used for histological evaluation in SCI animals 71, 72. This is necessary to be performed for SCI in the future. Studies have shown that besides over production of ROS, potassium efflux, and cathepsin B release can also cause NLRP3-induced pyroptosis 73, 74, and future studies should also explore whether BA influences K^+^ and CTSB in SCI. Finally, while these preclinical findings are promising, more work is needed to be done to explore optimal dosing and toxicities of BA in the treatment of SCI prior to clinical translation.

**Figure 8:**
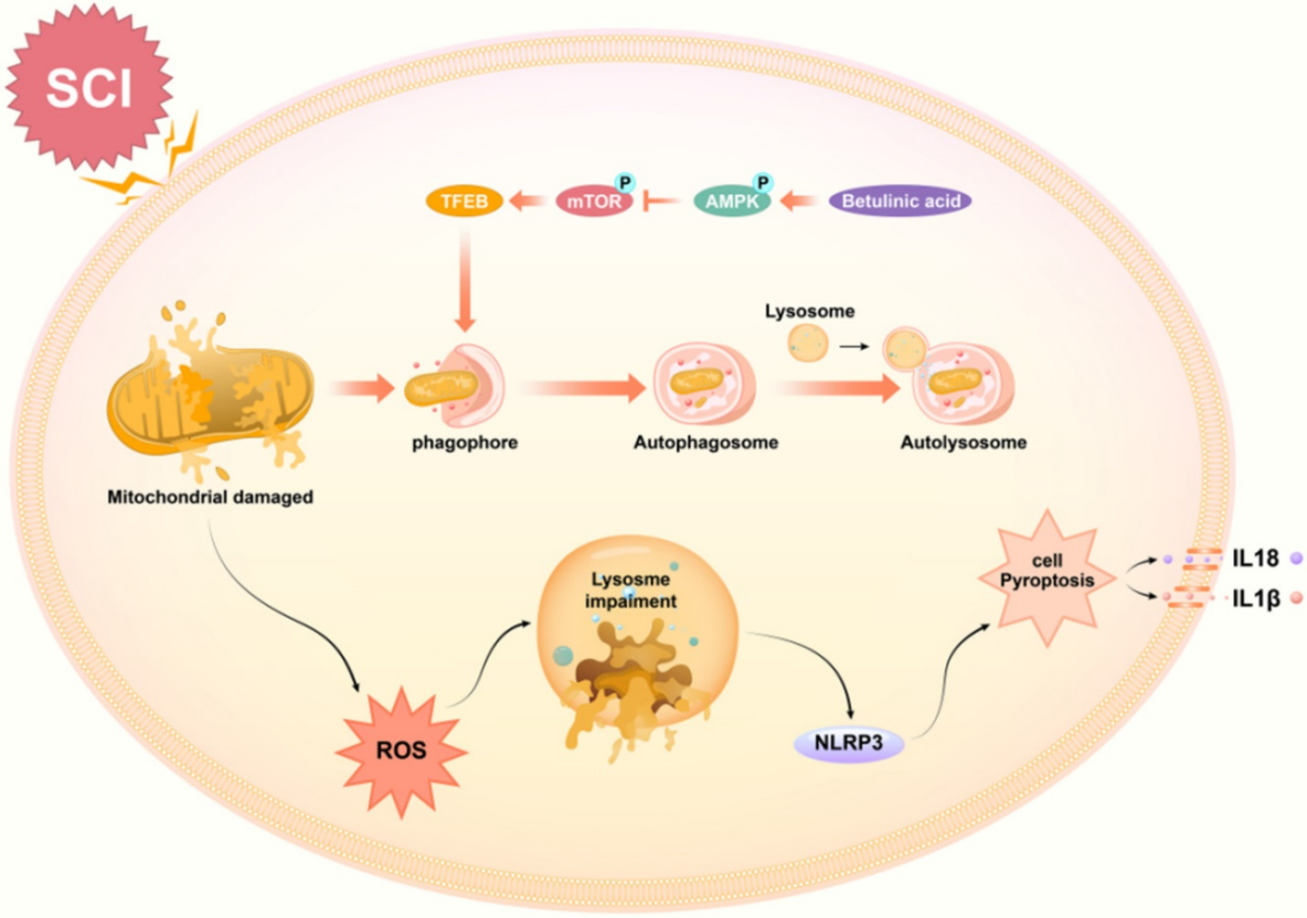
Schematic illustration of the proposed molecular mechanism highlighting the role of betulinic acid in the pathophysiology of SCI and subsequent neurological recovery. Betulinic acid activates autophagy through AMPK-mTOR-TFEB signaling pathway. Then enhanced autophagy and mitophagy to contribute to the elimination of damaged mitochondria and ROS by forming autolysosomes with lysosomes. Subsequently, pyroptosis are inhibited, which further results in the promotion of functional recovery after SCI.

Taken together, our findings demonstrate that BA activates the AMPK-mTOR-TFEB signaling pathway, which enhances autophagy in SCI. Increased autophagy induces mitophagy and reduces ROS accumulation, subsequently inhibiting pyroptosis. Ultimately, these effects of BA culminate in improved outcomes following SCI. A schematic illustration of our findings is presented in **Fig. 8**. Overall, these results provide novel preclinical evidence demonstrating the therapeutic benefit of BA in SCI. Future investigations are now eagerly awaited to further the clinical translation of BA as a treatment for SCI patients.
